# Neural mechanisms involved in female mate choice in invertebrates

**DOI:** 10.3389/fendo.2023.1291635

**Published:** 2024-01-10

**Authors:** Sagrario Cordero-Molina, Ingrid Fetter-Pruneda, Jorge Contreras-Garduño

**Affiliations:** ^1^ Laboratorio de Ecología Evolutiva. Escuela Nacional de Estudios Superiores. Universidad Nacional Autónoma de México, Ciudad de México, Mexico; ^2^ Departamento de Biología Celular y Fisiología, Instituto de Investigaciones Biomédicas, Universidad Nacional Autónoma de México, Ciudad de México, Mexico; ^3^ Institute for Evolution and Biodiversity, University of Münster, Münster, Germany

**Keywords:** mate choice, invertebrate brain, neural mechanisms, sexual selection, sexual conflict

## Abstract

Mate choice is a critical decision with direct implications for fitness. Although it has been recognized for over 150 years, our understanding of its underlying mechanisms is still limited. Most studies on mate choice focus on the evolutionary causes of behavior, with less attention given to the physiological and molecular mechanisms involved. This is especially true for invertebrates, where research on mate choice has largely focused on male behavior. This review summarizes the current state of knowledge on the neural, molecular and neurohormonal mechanisms of female choice in invertebrates, including behaviors before, during, and after copulation. We identify areas of research that have not been extensively explored in invertebrates, suggesting potential directions for future investigation. We hope that this review will stimulate further research in this area.

## 1 Introduction

**Figure 1:**
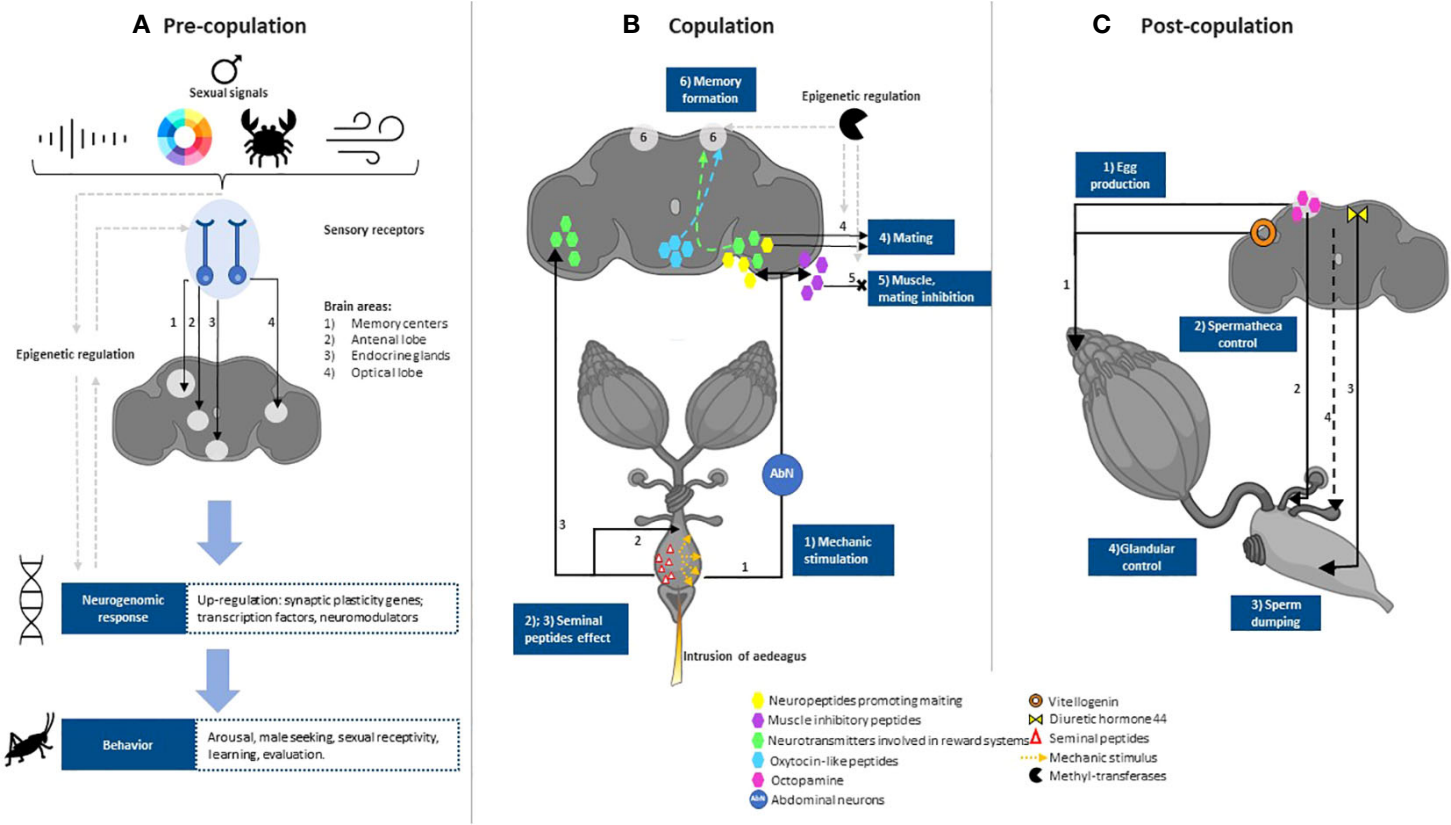
General description of neural pathways during female choice in insects. **(A)** Pre-copulation: Female mate choice is influenced by gene expression cascades that respond to stimuli from both the potential mate (e.g., sounds, colors, structures, pheromones) and the environment. The process begins with neural pathways that carry information from the sensory organs to integration points in the corresponding brain areas. There, synaptic plasticity genes activate primarily to produce suitable, plastic, and immediate responses based on the intensity of the signals presented by males (16, 17). If appropriate, such neural activity will prompt arousal, motivation, and reward systems mainly modulated by neurotransmitters (18). Furthermore, all the mentioned steps are subject to epigenetic regulation, while simultaneously being able to influence the same epigenetic regulators. Finally, the neural responses will result in behaviors that favor mating. **(B)** Copulation: During copulation, mechanical stimulation from the aedeagus (or pennis-like structure) intrusion stimulates abdominal neurons that carry the signal and triggers neural release of neurotransmitters involved in reward systems (18–20), such as dopamine (21). Motivation and reward systems facilitate sexual receptivity and copulation. The mechanical stimulus could inhibit re-mating through muscle inhibitor peptides or by blocking rewarding systems. In certain species, neurotransmitters may modulate pair bonding and memory formation mediated by oxytocin-like peptides in the brain (22). Changes in brain areas responsible for memory could trigger partner preference but may also lead to rejection of less attractive males or to former mates in the case of polyandrous females. In addition to mechanic stimulation, male ejaculate contains peptides that have the potential to act in the brain and release other neurotransmitters (23, 24). Other seminal peptides also have effects on the neural control of the female reproductive tract. Some of the neural process occurring during copula may be regulated by epigenetic enzymes, such as methyltransferases (17). **(C)** Post-copulation: Following copulation, additional mechanisms enable females to bias the paternity of their offspring or counteract male manipulation prior to or during copulation (25). Because the neural mechanisms of this stage are not well understood, a general description of what occurs in this stage is not yet possible. However, some works provide insights into the neural mechanisms of certain female behaviors. For example, hormones such as Diuretic hormone 44 in *Drosophila*, responsible for controlling neurons in the muscle of the female tract to control sperm retention or dump (26). Octopamine and octopamine receptors located in the decision-making brain areas of insects are required for ovulation, egg laying and muscular contraction of spermathecae (27–29). Vitellogenin is required for egg production and maturation, but is also located in the brain of social insects during reproduction and mate choice (17, 30). Females bias fertilization through sperm activation and/or deactivation, for example by glandular secretions that preserve the stored sperm (31) or with spermicidal action (32), it is unknown whether females exercise glandular control via neural actions from decision-making areas. Created with BioRender.com.

Mate choice is one of the most critical decisions organisms make because of its direct impact on their fitness. Mate choice is an evolutionary process that often favors the evolution of conspicuous traits in individuals (1). These traits, called secondary sexual characters (SSCs), include structures, colors, odors, and behavior (**Box 1**). Mate choice occurs when the evolution of SSCs in one sex leads to non-random mating with members of the opposite sex based on those characters (7, 8). Although mate choice was recognized over 150 years ago (3), many gaps exist in our understanding of its underlying mechanisms. For example, most studies on mate choice focus on the evolutionary causes of behavior, with less attention given to the physiological and molecular mechanisms involved (9, 10). Furthermore, although female choice is recognized as the more frequent type of mate choice (1, 11, 12), there have been more studies focusing on male behavior during the process of mate choice compared to female behavior (13). This bias may exist because male behaviors tend to be more conspicuous than female behaviors, resulting in a misinterpretation of females in a passive role in sexual selection. However, it is important to understand that the information conveyed by males through their behavior is interpreted within the female’s nervous system (14, 15) (**Figure 1**). Studying how female brains work during mate choice is crucial for fully understanding these behaviors from mechanism to evolutionary consequences.

Box 1 **Sexual selection.**

Darwin described sexual selection as “a struggle not for existence concerning other organic beings or external conditions, but a struggle between individuals of the same sex, usually males, for possession of the other sex” [(2), p. 59]. The result of this fight is not death but the lack of offspring of individuals. This implies that males with the most exaggerated secondary sexual characteristics (SSCs) (coloration, plumage, antlers, pheromones, etc.) will be favored by mate choice to leave more progeny. Sexual selection has three components. The first two, mate competition and mate choice were described by Darwin (3). The third, sexual conflict between mating pairs, occurs when males and females compete with their partners to obtain an optimum between the costs and benefits of mating and reproducing, with this optimum differing between the sexes (4–6).

While a growing number of studies in vertebrates reveal the neural mechanisms involved in the process of selecting a mate, our comprehension of the neural mechanisms of mate choice in invertebrates remains limited. DeAngelis and Hofmann reviewed the most relevant works in vertebrates on the neural and molecular mechanisms of female choice, focusing mainly on decision-making (9). Although such studies in invertebrates are limited, the existing research provides an opportunity to explore several important research avenues. 1) What are the specific locations and mechanisms within the nervous system that govern the intricate process of mate choice decision-making? and where in the brain does the process of mate choice occur: before, during, or after mating? 2) Given the diversity of mating strategies invertebrates possess (33, 34), is there a corresponding diversity in their neural mechanisms, or do a few mechanisms control such diversity? 3) Are the neural mechanisms underlying behaviors that are less explored or absent in invertebrates, such as selective use of stored sperm or sexual cannibalism, controlled by similar mechanisms to better studied behaviors in vertebrates? 4) The mechanistic understanding of mate choice behavior in invertebrates may provide hints to their origin and evolution, and this knowledge may be important for understanding the variety of ways that such behaviors can occur and may additionally allow a better understanding of the evolution and mechanisms of behaviors that we observe in vertebrates.

Here, we review the neural mechanisms of female choice in invertebrates during mate choice. We specifically include works that focus on the role of genes, molecules (such as neurotransmitters, hormones, and proteins), and neurons that participate in female choice. We consider female choice to be a behavior that contributes to non-random mating and/or fertilization, including behaviors before, during, and after copulation. Furthermore, we identify areas of research that have not been extensively explored in invertebrates, suggesting potential directions for future investigation.

## 2 Precopulatory neural mechanisms

A crucial early phase in mate choice involves the accurate perception of signals from a potential mate, with a primary function being the identification of the sender as a member of the opposite sex (1). The mechanisms behind this stage will depend mainly on the nature of the signals and the type of organs and sensory cells used to receive the stimulus, e.g. chemoreceptors in the antennae of fruit flies (35, 36), the cuticle of nematodes (37, 38) or the ventral mesosoma of scorpions (39); the mechanoreceptors of *Drosophila* (40), crickets (41) and arachnids (39, 42); UV sensitive and other wavelength photoreceptors in odonates (43) and spiders (44, 45). The type of male signals will determine the neurobiological pathway that will carry this information to the integration centers in the female brain (**Box 1**) (46, 47). After the individual has been identified as a male of the same species, the female will be able to discriminate between potential mates.

### 2.1 Sexual receptivity

Before choosing a male, the sexual status of the female determines her receptivity to copulation. A receptive female often displays a series of behaviors facilitating reproduction (48), which may include the release of chemical signals, movements toward males, or ovipositor extrusion. The neural mechanisms for sexual receptivity have been studied in *Drosophila melanogaster*. When immature virgin or recently mated *D. melanogaster* females are not receptive, they do not respond to the male’s courtship and instead avoid him by performing specific stereotypical movements: immature virgins curl their abdomen tip downwards, a behavior referred to as “curling”; mated females and immature virgins engage in a behavior called “decamping”, which involves running, jumping, or flying away; and mature females and immature virgins kick backward, a behavior termed “kicking”. (49) When receptive, however, they pause their movements and interact with male partners to copulate, a behavior referred to as “pausing” (18, 49). Two primary genes regulate *D. melanogaster*’s receptivity, *doublesex* (*dsx*) and *fruitless* (*fru*). Both are transcription factors responsible for differences in male and female sexual behaviors, and these differences are also observed in the neuronal circuits (50–53). The description of the neural circuitry of sexual receptivity in *Drosophila* has allowed the identification of *dsx*- and *fru-expressing neurons*, dendritic projections, and connection sites in the brain and reproductive tract (18, 52, 53). Zhou et al. found that the silencing of *dsx-expressing* neurons reduced female receptivity (53). In addition to *dsx*, Bussell et al. showed that the transcription factor Abdominal-B (Abd-B) regulates the female behavior of pausing in front of courting males and interacting with them to copulate (18). Abd-B is present in a group of neurons in the abdominal ganglion with projections to the brain and reproductive tract, which suggests that their function is to integrate the sensory information of courtship and output the observed motor activity, such as “pausing” (18). The nervous system of females can be altered by peptides produced by themselves, but also by those transferred by males in the spermatic fluid (23). As an example, the sex peptide (SP) is originated in the male accessory glands and transferred during insemination to females. SP elicits alterations in neural activity that reduce receptivity (23). Within the female’s reproductive tract, sensory neurons responsive to sex peptide detect its presence and convey the signal to the SP-abdominal ganglia neurons and to myoinhibitory peptide interneurons. Concurrently, SP suppresses the activity of serotonergic projection neurons, leading to a decline in female receptivity (54–57).

### 2.2 Neural response to male courtship

The neural circuits of sexual behavior in invertebrates have been primarily studied in *Drosophila*. Immonen and Ritchie identified differences in gene expression in *Drosophila simulans* female brains exposed to courtship songs by conspecific and heterospecific males (36). They identified several antennal signaling genes that are part of neuropeptide signaling pathways, including the neuropeptide Corazonin (Crz) (36) which is involved in dopamine regulation and modulates female sexual receptivity in *Drosophila* (21). In addition, they found that exposure to courtship songs influences the expression of genes involved in chemical communication, such as odorant receptor and co-receptor genes and odorant-binding protein-coding genes, most of which are involved in binding pheromones (36). These findings suggest that male courtship sensorial stimulation facilitates receptivity in females by enhancing their sensitivity to male chemical signals.

Female response to courtship may change after mating, as evidenced by olfactometry studies that demonstrate a decreased response to male olfactory cues in mated females of *Anastrepha* fruit flies compared to their unmated counterparts (58). While the neural pathways governing female responses to male courtship have been extensively elucidated in *Drosophila*, studies on other invertebrate species have identified specific components involved in responding to various types of male signals, including visual and vibrational stimuli (conveyed through leg and pedipalp drumming) in the courtship of *Schizocosa* wolf spiders (42). Notably, *Schizocosa* females possess vibration receptor organs in their legs comprised of multiple slit sensilla, each innervated by two neurons (42). Research by Knowlton and Gaffin (42) revealed that neurons in all sensilla, both proximal and distal, responded to leg drumming, with proximal sensilla exhibiting a higher response to pedipalpal drumming. It’s worth noting that pedipalps are also employed for sperm transfer. Therefore, the diverse responses to vibrational signals from different sources provide clues into the precision and sensitivity involved in detecting signals during courtship. Studying the nervous system during courtship not only enhances our understanding of female responses but also helps determine more precisely which male signals elicit a response in the female. Recent evidence suggests that, during courtship, the leg movements of male *Schizocosa retrorsa* induce air particle movements, which females respond to during mate choice, rather than responding solely to the visual stimulus (59).

### 2.3 Neural responses during mate evaluation

Experience with different males causes changes in female brain activity and affects how they choose a partner. Experience and exposure to males with variation in their SSCs play an essential role in how females choose in future encounters. In flies and crickets, it has been observed that females increase the acceptance threshold of potential partners according to the type of males with whom they have had previous social experiences (60, 61). Although the mechanisms behind the increase in choice threshold remain unknown, one suggestion is that *Drosophila* females become more selective after mating because copulation increases their Juvenile Hormone (JH) levels, which reduces olfactory sensitivity of Or47b odorant receptor neurons that sense the male sex pheromone (62). Therefore, in a circumstance where a previously mated female encounters more than one male, the chosen male will be the one that produces enough sex pheromones to surpass the threshold, which could lead to strong intra and intersexual competition.

Sensory modalities in mate choice may vary across species. However, other processes involved in information integration and neural response modulation may be evolutionarily conserved. For instance, the process of mate choice in vertebrates includes alterations in gene expression related to neural plasticity (16, 63), along with epigenetic control (64). Hernandez-Villanueva et al. found similar responses in the brain of *Tenebrio molitor* females, consisting in an increase in proteins related to synaptic plasticity and changes in the levels of methyltransferases and subunits of the methyltransferase complexes when they evaluate and mate with males of different phenotypes (17).

### 2.4 Attention-directed behaviors of sexual signals from various males

During female choice, females receive male signals from various potential partners and should differentiate one signal from another to choose the best option, that is, the signal that favors their fitness. This could be challenging in systems where several males court females simultaneously. In these cases, the female must be able to differentiate the signals of potential partners and focus her attention only on one male while ignoring the others. In crickets (*Mecopoda elongata*), when females receive acoustic signals from several males that give similar calls at brief time intervals, they prefer the leading male, who generally sets the interval rate that other males follow (65). Omega neuron 1 (ON1) neurons are involved in perceiving acoustic signals and receive excitatory monosynaptic connections from ipsilateral afferent sensory cells. ON1 neurons also have inhibitory activity on their contralateral connections (41). When females receive identical signals from two males in opposite directions, the ON1 neurons that receive the signal from the leading male are excited and simultaneously inhibit the contralateral ON1 neurons, preventing them from perceiving the other males’ acoustic signals (41). This raises the question of how the female brain discerns between males in species in which males court females as a group (lek) and how these mechanisms vary in subsequent encounters.

## 3 Copulatory neural mechanisms

A species’ mating system can be classified as either monogamous or polygamous (**Box 2**) (66), which may differ in their copulatory mechanisms. However, there is currently a gap in our knowledge, as molecular studies investigating gene expression and neural changes in the female brain of invertebrates with different mating systems are notably lacking, and potential behavioral disparities between these mating systems may be rooted in variations in the neural mechanisms that drive frequent mating with one or more partners. This information is also essential for determining whether these mechanisms are analogous to those found in vertebrates [e.g. (69) and (70)]. Furthermore, females may influence the outcome of copulation in several ways, which may vary by mating system. Females may respond positively or negatively to copulatory stimulation, they may control the duration of copulation (71) or may bias sperm utilization by favoring certain males (72, 73).

Box 2 **Mating systems.**

A mating system refers to the way in which individuals within species establish and maintain sexual pairs, as well as how fertilizations are achieved and which individuals are involved (66, 67). These systems are frequently influenced by a range of factors, encompassing ecological, social, and evolutionary aspects. Studying mating systems is invaluable for comprehending the reproductive strategies of diverse animal species and the dynamics of their populations. In the animal kingdom, various mating systems are observed, and they can be broadly classified into the following main types (66–68):

Monogamy: In a monogamous mating system, a single male and a single female form a long-term (often lifelong) bond. Monogamy ensures that both parents participate in the care and protection of their offspring. When females exhibit this behavior, it is referred to as monandry.

Polygamy: Polygamy is a mating system where both sexes mates with multiple individuals of the opposite sex. When females display this type of behavior, the term used is polyandry.

Promiscuity: In a promiscuous mating system, individuals have multiple, often brief, sexual encounters with multiple partners, without forming long-term bonds. This is common in many species, including many insects and some mammals.

### 3.1 Responses to stimulation during copulation

In vertebrates, there is ample evidence that mechanical stimuli received during copulation can exert changes in the neuronal response of the female reproductive tract (74–76). In *Drosophila*, during copulation, intrusion by the male aedeagus has been shown to decrease the activity of neuron clusters in the central brain [possibly pC1 and pCd populations (53)], resulting in reduced sexual receptivity of females independent of the action of seminal molecules that males transfer (19, 20). Female abdominal neurons expressing Piezo mechanosensitive channels are stimulated by insertion of the aedeagus. Then, the signal is transmitted to ascending neurons called LSANs that connect in the central brain (20). Shao et al. proposed that LSAN neurons connect to other neurons that produce a peptide known as muscle-inhibitory peptide (also described as allatostatin-B in *Drosophila*) resulting in a reduction in female receptivity (20).

### 3.2 Control of copulation duration

Duration of copulation is an important factor in how much ejaculate is transferred from the male and how likely fertilization is to occur. Abdominal ganglia are heavily involved in regulating muscle contractions in the reproductive tract to influence copulation duration (20). Although the specific neuronal centers involved remain unidentified, the brain also plays a significant role, as supported by observations in three fly species [*Musca domestica* (77), *Anastrepha suspensa* (78), and *Batrocera tryoni* (71)] that copulation with decapitated or decerebrated females lasted longer than with intact females (71, 77, 78).

### 3.3 Monogamy: neuromodulatory mechanisms of pair bonding

After sexual attraction and choosing a mating partner, some species may form pair bonds characterized by selective attachments with some degree of durability (70, 79). Much of the knowledge about the neural mechanisms of pair bonding has been generated through the study of strictly monogamous species, mainly mammals such as the rodent *Microtus ochrogaster* (70, 79, 80). In this species, the peptides oxytocin and vasopressin modulate pair bonding (79, 80), and together with the dopaminergic system in the brain, promote continuous mating with the same partner (70). It has been proposed that the genes encoding oxytocin and vasopressin originated about 600 million years ago (81), with the predecessor molecules to these peptides having a similar role as modulators of social behaviors in different taxa (82–86), including invertebrates (87). However, the role of oxytocin-like peptides during mate choice in invertebrates has yet to be explored, likely because they are absent in flies and bees, precluding their study in honey bees, the most widely studied social insect, and *Drosophila* (88). Most descriptions of the role of oxytocin-like peptides are about behaviors associated with male copulation in nematodes (88), hirudines (89), and gastropods (90, 91). However, its role in female pair bonding and copulation is poorly understood.

Some invertebrate species are described as monogamous, remaining with a single sexual partner for an extended period of time, usually marked by the period of parental care, or because they mate only once in their lives (92). Monogamous behavior and its neural mechanisms have been studied in the biparental beetle *Lethrus apterus*. In this species the expression of genes encoding the insect oxytocin-like peptide, inotocin (*int*), and its receptor (*intr*) in the brain increase during reproductive season and are highest at the beginning of pair formation and during the period of parental care (22). These findings suggest that, as observed with oxytocin in vertebrates, inotocin could modulate temporary mate-attachment behavior in insects. More research is needed on the role of inotocin in species typically described as monogamous or performing biparental care. Additionally, while it is intuitive to study monogamy in species that are commonly considered monogamous or exhibit biparental care, the mating systems in invertebrates are much more complex and flexible than typical descriptions found in other species. Excluding these models limits our understanding of how different mechanisms have evolved in similar behaviors. For example, in *Gonodactylus bredini* shrimps, it has been observed that males and females reduce their aggression towards their former mates when they meet again (93). Some shrimps of the genus *Alpheus* are considered socially monogamous (pair bond without implying sexual exclusivity between the two partners) since both sexes defend the territory and sometimes provide the nest with food, benefiting more from living in pairs than alone (94). Many molecular pathways can be conserved over long periods of evolutionary time, and understanding what molecules are involved in the modulation of mate attachment of invertebrates would allow a better understanding of the evolution of the molecular systems behind pair bonding that have been widely described in typically monogamous mammals and other socially monogamous vertebrates.

In addition to oxytocin-like peptides, other neuropeptides may be involved in regulating mate attachment. Cunningham et al. reported several neuropeptides that orchestrate biparental care in the beetle *Nicrophorus vespilloides* (95). For example, Natalisin FMRFamide and Sulfokinin have functions that promote mating in various taxa (96–98); Tachykinin is involved in aggression (99); neuropeptide-like precursor 1 (NPLP-1) is involved in the division of social labor in honeybee workers (100); and Pheromone-Biosynthesis-Activating Neuropeptide (PBAN) activates pheromone synthesis (101). These neuropeptides might be important for pair bonding because biparental care is one of the most important selective pressures for the evolution of monogamy (66), and in *Nicrophorus vespilloides* pair bonding and parental care of the larvae are long-lasting.

#### 3.3.1 Other molecules involved in social interactions and pair bonding

Another molecule related to insect social behavior is vitellogenin (Vg), whose primary known function is the production of yolk proteins in oviparous animals (102). Nevertheless, Vg has also been detected in the brains of social insects such as *Apis mellifera* (103) and *N. vespilloides* (30); as well as in the brains of female *Tenebrio molitor*, a polygamous species (17). Vitellogenin is also linked to parental care, with expression of it and its receptor decreasing during active parental care and varying throughout the reproductive cycle in both sexes of the subsocial beetle *Nicrophorus vespilloides* (30), and to the regulation of genes in the brain that are involved in division of labor in eusocial insects, such as insulin receptor precursor, JH epoxide hydrolase, Impl3 (ecdysone-inducible gene L3); PLRP2 (pancreatic lipase-related protein 2 precursor); Sirt6 (sirt 6 histone deacetylase); TRIP4 (thyroid hormone receptor interactor 4), the transcription factor fruitless in the brain (104, 105). The relationship between Vg expression in the brain and social behavior is not yet clear, but it is also known to function in the brain to regulate energy metabolism of glial cells (103) and to buffer against damage and oxidative stress (106), which may provide some clues. Memory and decision-making demand a large energy expenditure in the brain (107), and cognitive processes such as mate recognition may have a high metabolic demand. A potential area for future investigation could involve examining whether vitellogenin mitigates brain damage in species where females remember previous partners or engage in continuous partner assessment based on previous experiences with males. An example of this possibility is observed in *T. molitor* where vitellogenin levels were higher in the brains of females that evaluated more attractive males compared to those that evaluated less attractive males (17). Furthermore, females exposed to attractive males exhibited elevated levels of catalase in comparison to those interacting with non-attractive males (17). The increase in catalase levels implies the activation of an antioxidant defense mechanism within the brain. Whether the high metabolic rate in females evaluating different phenotypic males is connected to such a mechanism remains to be answered.

Evidence shows that the peptides and proteins mentioned in the above section are important candidate molecules to study their role as regulators of social behavior during mate choice in invertebrates. However, most approaches focus on analyzing brain gene expression during mate choice, pending behavioral observation when these molecules’ functions are altered, for example, through silencing or using agonist and antagonist drugs. Combining different experimental approaches would allow knowing if the selected molecules or genes are the only effectors of the behaviors mentioned or if it is a behavior that responds to more than one effector.

### 3.4 Neural mechanisms promoting polyandry

Polyandry is the system where a female mates with more than one partner (66). However, many mating systems are flexible, with females able to undergo phases of monandry or polyandry in the face of changing ecological contexts (108), such as the availability of mates in some schistosome species (109) and the damselfly *Ischnura hastata* (110); or environment resources as seen in the beetle *Ips latidens* (111) and the butterfly *Pieris napi* (112). In addition to the absence of mate recognition and attachment systems described in monogamy, the neural mechanisms of polyandry should address; 1) the motivation to seek and accept copulation with multiple males, and 2) behaviors promoting copulation with new males, such as aggression towards previous partners with the intention of increasing offspring variability (66). Re-mating motivation mechanisms usually involve the same genes and neurons as receptivity mechanisms (79, 113), and differences between mating systems might be due to different activational states of these circuits.

In addition to signals perceived before copulation, seminal proteins and peptides can affect female sexual receptivity circuitry (23, 54, 114), leading the female to change temporarily from polyandry to monandry or vice versa. In the cricket *Teleogryllus oceanicus*, the seminal proteins ToSfp022 and ToSfp0 appear to be responsible for reducing mate-seeking behavior, as females mated with males with a knockdown in the ToSfp022 and ToSfp01 genes left their nest significantly more frequently in response to male courtship songs compared to those mated with control males (24). While the mechanism of action of these proteins remains unclear, evidence suggests they interact with the female nervous system in a manner similar to sex peptide in *Drosophila* (23, 54, 57).

Finally, among the benefits of polyandry are increased genetic variability of the offspring and/or receiving more direct benefits from different males [i.e. nuptial gifts, nutrients and high sperm reserves (115, 116)]. Therefore, in studying the neural mechanisms of polyandry, it is essential to include the behaviors of rejection and aggression toward known males with whom they have previously mated. Such behaviors are described in different taxa, such as the pseudoscorpion *Cordylochernes scorpioides* (117), the spider *Pholcus phalangioides* (118), the moth *Ephestia kuehniella* (119), and the cricket *Gryllodes sigillatus* (120). Although for now this explanation remains hypothetical, these works suggest that females recognize their previous partners through chemical signals that they transfer to males during copulation. These chemical cues might trigger changes in female gene expression that could inactivate neural circuitry for sexual receptivity upon subsequent encounters.

## 4 Postcopulatory neural mechanisms

Females can skew the paternity of their offspring during and after copulation, a phenomenon called cryptic female choice (25, 121). The mechanisms involved include morphological traits in the reproductive tract or genitalia, physiology, and behaviors that non-randomly favor paternity in certain males (122, 123). Some behaviors include control of copulation duration, retention, or expulsion of the ejaculate, and cryptic elimination of spermatozoa by substances with spermicidal action (25). Below we describe neural mechanisms studied during cryptic female choice in invertebrates.

### 4.1 Neural control of spermathecal contraction

Female arthropods have sperm storage organs called spermathecae in which the sperm of one or several males can be stored for use long after copulation has finished. This results in males competing against the sperm of their rivals inside the female’s reproductive tract (sperm competition). Based on the neural control of the spermatheca by muscles in insects (124, 125), it has been suggested that females could actively bias fertilization through the nervous control of the spermatheca (27, 123, 126–128). Although it is not yet clear how the spermatheca might bias male paternity, important advances have been made in studying the mechanisms that control spermatheca contractions in some insects. In *Locusta migratoria*, females release sperm stored in the spermatheca through contractions that begin when sensory cells located on the wall of the genital chamber are activated by mechanical stimulation from passage of the egg to be fertilized (129, 130). Joint action of octopamine and tyramine increases the frequency of muscular contractions of the spermatheca in *D. melanogaster* (123) and in *L. migratoria* (28, 131). Furthermore, in *D. melanogaster*, octopamine receptors in the mushroom bodies (OAMB) are required both for ovulation (29) and the release of sperm from spermatheca (123). In *D. melanogaster*, these results imply that the mushroom bodies are involved in controlling reproduction and ovulation. This makes sense with the fact that the mushroom bodies are the primary information integration centers in the insect brain, where learning processes, memory, odor, and size discrimination occur (132–134). These results support the idea that females may bias the paternity of some males based on cues received from male evaluation before or during copulation.

### 4.2 Sperm dumping

Another behavior by which females may bias male paternity is the selective expulsion of sperm that the male has transferred to her. The neurobiological pathway behind this behavior has been studied in *D. melanogaster* through the dynamics of Diuretic Hormone 44 (DH44) and its receptor Diuretic Hormone 44 Receptor 1 (DH44R1) in the brain. By silencing transcription of the genes encoding DH44 and its receptor in neurons of the pars intercerebralis region with RNA interference, sperm expulsion occurred much more rapidly than in control females (26). The *dh44* and *dh44r1* genes are orthologous to the *corticotropin-releasing factor* (*crf*) and *corticotropin-releasing factor receptor* (*crfr*) genes that have stress response functions in vertebrates (135). Therefore, the authors suggested that sperm expulsion in *Drosophila* could be a stress response caused by the seminal peptides transferred during copulation (26, 136).

In addition to the possible effect of seminal peptides, control of sperm expulsion may occur based on the perceived genetic quality of the male before or during copulation as seen in females of the spider *Physocyclus globosus*. In this species, sperm expulsion occurs during or after mating, but females favor paternity for males who display increased courtship behaviors before and during copulation (73). The relationship between courtship intensity and sperm expulsion suggests the possibility that the neural pathways regulating sperm retention are affected by molecules and circuits implicated during courtship or copulation. Such neural pathways could be connected to reward circuits involving dopamine, which may regulate female decisions during mate choice either by promoting mate search and/or acceptance of mating after evaluation of males (137). Neural circuits promoting mating and courtship in male *Drosophila* have been identified, but female neural responses to courting males are poorly studied. Nonetheless, the transition from rejection to mate acceptance in virgin females may be controlled by the ellipsoid body (EB), a structure of the central complex (138). The interconnected ring neurons (R) in the EB receive input from the PPM3 clustered dopaminergic neurons situated in the superior medial protocerebrum. In the EB, Cholinergic R4d neurons promote rejection behaviors, while the activation of GABAergic and glutamatergic R2/R4m neurons promotes mating acceptance. Additionally, inhibiting R2/R4m neurons leads to an increase in mating latency (138, 139). Although this finding aligns with reports in various vertebrate dopaminergic circuits (137), further research on female neural responses is crucial and may be fruitful considering the possibilities for manipulating the *Drosophila* system (138, 139).

### 4.3 Control of oviposition

Even after fertilization has occurred, it is possible that females exert some control of oviposition, influencing which eggs are laid (121). Octopamine regulates oviposition in insects (140–142), ticks (143) and nematodes (144, 145) by controlling the contraction rhythms of the oviduct. In *Drosophila*, the Octb2R and OAMB receptors present in octopaminergic and glutamatergic neurons in the abdominal ganglia project to the epithelial and reproductive cells of the oviducts (29, 142, 146). Recent studies indicate that activation of the Octb2R receptor causes relaxation, while activation of the OAMB receptor causes contraction (146). Additional neuromodulators, such as glutamate, tyramine, and other biogenic amines, may alter the impact of octopamine on the aforementioned neurons. However, their effects remain poorly comprehended and may vary among species (140–146).

### 4.4 Other behaviors of postcopulatory female choice

There are several exciting behaviors to study in postcopulatory female choice for which there is not yet an approach to study the possible neural mechanisms behind them, for example:

Activation and inactivation of spermatozoa. In several invertebrate species, females may influence fertilization through substances secreted in their reproductive tract that either activate or disable sperm transferred by their partners. For instance, the female reproductive tract of *Drosophila pseudoobscura* provides a spermicidal environment that contributes to sperm competition (32). Another intriguing example comes from spiders, where glandular secretions activate and maintain the sperm stored within spermathecae (31, 147). It has been proposed that the female nervous system regulates the secretion of these glands (148), which may act as a mechanism to bias fertilization towards preferred males, such as those with higher courtship intensity (149).

Selective uptake of sperm in the spermatheca. Paternity may be biased by controlling the reception of sperm in the spermatheca (25). There is some research into the mechanism involved in the release of sperm stored in the spermatheca, but the mechanism controlling the uptake of sperm into the spermatheca remains unknown.

## 5 Female choice issues that have yet to be explored in invertebrates

### 5.1 Sexual cannibalism

One of the most extravagant behaviors in sexual selection is sexual cannibalism, when the female consumes the partner during or after mating and sometimes during courtship without mating. This behavior has only been documented in spiders and mantids. Sexual cannibalism might increase male reproductive success and, in many cases, results from female choice based on the quality of their partners (31, 150, 151). The neural mechanisms of this behavior are yet to be addressed. Based on descriptions of the behavior, it can be hypothesized that the visual sensory system as well as circuits and systems related to foraging and aggression are involved (31), but how these systems relate to mate choice is an open question.

Another example of sexual cannibalism is the mutual “partial” cannibalism of the cockroach *Salganea taiwanensis*. In this monogamous species, both sexes reciprocally consume the wings of their mate. The hypotheses for the evolutionary causes of this behavior propose that consumption of the wings promotes monogamy since, without wings, it is dangerous to leave the nest to look for another mate (152). Because this type of cannibalism promotes pair bonding and does not end in complete consumption of the partner, the neural mechanisms behind this behavior may differ from the aggression mechanisms proposed for sexual cannibalism in spiders and mantids.

### 5.2 Female neural responses to male manipulation

Sexual conflict arises when some of the following circumstances are in place (reviewed in: 147): 1) males try to overcome filters imposed by the females during mate choice, while the females respond with counter-adaptations to male strategies (153); 2) males exert sensory exploitation of pre-existing circuits in females (154); 3) females resist exploitation or manipulation by males (154, 155). However, sexual conflict is mostly detected through observation of behavior, and most of the conclusions do not address how the female’s nervous system responds to male manipulation. The investigation of the neural responses of females during sexual conflict would advance the comprehension of how sexual selection is taking place across species. For instance, in species where male manipulation occurs, analyzing behavior and reproductive success may not reflect females’ resistance to manipulation. However, looking through the nervous system of females could offer a different perspective. By exploring their neural responses, a “physiological attempt” of resistance may be revealed. While these responses are not yet considered counter-adaptive mechanisms, they have an evolutionary potential to develop and empower females to confront manipulation.

Some of the male manipulative behaviors are truly extreme, and studies on the neural responses of females when they occur are limited. One species where such a conflict occurs is the true bug *Gerris gracilicornis*. In this species, males attract predators via special leg movements when females they attempt to mount refuse their advances, leading to coercive copulation (156). How females decide to allow or deny copulation in the context of potentially being preyed on, and if the mechanisms of aggression and flight are coordinated and connected to those of sexual receptivity are interesting questions. Another extreme case of sexual conflict during mating is the traumatic insemination observed in the bed bug *Cimex lectularius*. In this species, males pierce the cuticle of females and inseminate them directly into their body cavity without penetrating their genitalia (157, 158). It is unknown whether the circuits connecting the female genitalia to the nervous system are active or inactive in such cases, or if other circuits are involved during insemination or when sperm is released from their storage organs. Comparison of this behavior with closely related species that do not display extra-genital copulation may provide insight into whether and how the neural response of these females to this type of male behavior has evolved.

In ants of the species *Hypoponera opacior*, some males mate with young nestmate queens even before they eclose from their cocoons (159, 160). It would be interesting to study any behavioral response to this in either the inseminated queens as pupae or post eclosion or in their egg-laying mother.

Mating plugs are another example of male manipulation, and it is not known what happens inside female brains when a mating plug prevents copulation with subsequent males in some species of arachnids (161–163); insects (164); and even nematodes (165). A mating plug could do more than just physically block copulation; it could also mechanically stimulate neurons of the reproductive tract resulting in inhibition of receptivity (166). Mating plugs are also utilized by male vertebrates (167, 168), however, research on the neural responses of females to this behavior is scarce.

### 5.3 Possible costs of neural processes of female choice

Sexual differences in reproductive strategies are usually explained as arising from different reproductive costs for each sex (5, 169), but could this same argument be applied to neural mechanisms? In numerous species, females select mates using a cognitive process that may incur ecological costs (17, 107, 170, 171); however, males may not face equivalent consequences because of the absence of neural elements that enable females to be more selective in their mates. Nevertheless, if there is male choice, they too may experience comparable costs. One such cost is intoxicating male sperm in *Drosophila* females (172, 173). In this case, the role of the female detoxification systems is unknown. It is certainly possible to address these questions in vertebrates, but simpler nervous systems, shorter generation times and faster metabolic rates may make invertebrates better candidates.

## 4 Conclusions

Study of the neural mechanisms of mate choice is essential for a comprehensive understanding of the unusual behaviors we observe in nature. There are an increasing number of studies on the neural mechanisms of mate choice. However, those on female choice are less common and usually have a broad descriptive approach and not under the theoretical framework of sexual selection. Females of various species display a broad diversity of mate choice mechanisms, and it is possible that this variety in mechanisms corresponds to variation in mating system, life history and evolutionary history. For this reason, it is essential that models used to study female mate choice are equally diverse.

As with other social behaviors, the mechanisms behind female choice are complex and difficult to study because several often occur simultaneously. As techniques, including single-cell sequencing, genome and epigenome sequencing, proteomics, and access to invertebrate genomes advance, it will be possible to disentangle mate choice behaviors with careful experimental design. Working together to understand the immediate causes behind the behaviors will complete the picture of the evolution of mate choice.

## Author contributions

SC-M: Writing – original draft, Writing – review & editing, Conceptualization. IF-P: Funding acquisition, Supervision, Writing – review & editing. JC-G: Supervision, Writing – review & editing.
